# A nomogram model for predicting distant metastasis of newly diagnosed colorectal cancer based on clinical features

**DOI:** 10.3389/fonc.2023.1186298

**Published:** 2023-06-15

**Authors:** Jiang-Hua He, Cong Cao, Yang Ding, Yun Yi, Yu-Qing Lv, Chun Wang, Ying Chang

**Affiliations:** ^1^ Department of Gastroenterology, Zhongnan Hospital of Wuhan University, Wuhan, China; ^2^ Department of Colorectal Surgery, Gansu Provincial People’s Hospital, Gansu, China

**Keywords:** colorectal cancer, distant metastasis, prognosis, nomogram, platelet count

## Abstract

**Objective:**

Colorectal cancer is one of the most common primary malignancies and the third most common cause of cancer death in both men and women in the United States. Among people diagnosed with initial colorectal cancer, 22% had metastatic colorectal cancer, while the 5-year survival rate was less than 20%. The purpose of this study is to develop a nomogram for predicting distant metastasis in newly diagnosed colorectal cancer patients and to identify high-risk groups.

**Methods:**

We retrospectively reviewed the data of patients who were diagnosed with colorectal cancer at Zhong nan Hospital of Wuhan University and People’s Hospital of Gansu Province between January 2016 and December 2021. Risk predictors for distant metastasis from colorectal patients were determined by the univariate and multivariate logistic regression analyses. Nomograms were developed to predict the probabilities of distant metastatic sites of colorectal cancer patients and evaluated by calibration curves, receiver operating characteristic curves, and decision curve analysis (DCA).

**Results:**

A total of 327 cases were included in this study: 224 colorectal cancer patients from Zhong nan Hospital of Wuhan University were incorporated into the training set, and 103 colorectal cancer patients from Gansu Provincial People’s Hospital were incorporated into the testing set. By univariate logistic regression analysis, platelet (PLT) level (*p* = 0.009), carcinoembryonic antigen (CEA) level (*p* = 0.032), histological grade (*p* < 0.001), colorectal cancer tumor markers (*p* < 0.001), N stage (*p* < 0.001), and tumor site (*p* = 0.005) were associated with distant metastasis in colorectal cancer patients. Multivariate logistic regression analysis showed that N stage (*p* < 0.001), histological grade (*p* = 0.026), and colorectal cancer markers (*p* < 0.001) were independent predictors of distant metastasis in patients initially diagnosed with colorectal cancer. The above six risk factors were used to predict distant metastasis of newly diagnosed colorectal cancer. The C-indexes for the prediction of the nomogram were 0.902 (95% confidence interval (CI), 0.857–0.948).

**Conclusion:**

The nomogram showed excellent accuracy in predicting distant metastatic sites, and clinical utility may facilitate clinical decision-making.

## Introduction

Colorectal cancer (CRC) is one of the most common primary malignant tumors and the third most common cause of cancer death in both men and women in the United States. In 2021, an estimated 1479,500 new cases and 52,980 deaths were projected in the United States (1). Among people diagnosed with initial colorectal cancer, 22% have metastatic CRC. Over the past 30 years, the incidence and overall survival (OS) rate of CRC have seen a significant improvement. The 5-year relative survival rate of CRC patients was approximately 65.1%. Although the prognosis of metastatic CRC is poor, with a 5-year survival rate of less than 20% (2, Accessed July 31, 2022), the survival rate has greatly improved because of the development of diagnosis and treatment schemes.

Metastatic CRC is defined as a metastatic disease or cancer that has spread beyond the original colorectal mass. The most common sites of distant metastasis include the liver, lung, and peritoneum (3). Many large sample studies (4–6) reported the cumulative metastatic rates of colorectal cancer in the liver (40% –50%), lung (10% –20%), and peritoneum (4%). Headways in the treatment of metastatic diseases, including improved surgical techniques, increased cancer-directed surgery, advances in the treatment of liver metastases, and the development of targeted therapies, are evident in survival gains for these patients in recent decades (3). It is clinically significant to detect distant metastasis (DM) in newly diagnosed CRC patients because early identification can help optimize treatment and management to increase the 5-year relative survival rate and quality of life.

In clinical practice, computed tomography (CT) is the most commonly used imaging examination to evaluate distant metastases of colorectal cancer patients. However, studies have reported that CT has a sensitivity of 65%–95% for colorectal cancer liver metastases with a diameter ≥ 1 cm, while it has a sensitivity of only 31%–38% for lesions with a diameter <1 cm, and the sensitivity further decreases if the patient has fatty liver (7). Recently, machine learning algorithms have played an important role in evaluating the metastasis and prognosis of malignant tumors. In gastric cancer, the literature reported seven machine learning algorithms to predict distant metastasis models, including logistic regression, random forest (RF), least absolute shrinkage and selection operator (LASSO) regression, support vector machine, k- nearest neighbor, naive Bayes model, and artificial neural network (8). David’s research used 11 machine learning algorithms to predict the short- and long-term survival probability of CRC patients (9).

In the previous studies, many risk factors and prognostic variables were identified, including tumor markers, histological type, tumor location, platelet count (10), and tumor–node–metastasis (TNM) staging system. These factors are related to the prognosis of colorectal tumors (11). The prognosis of CRC patients varies in different clinicopathological factors, especially for colorectal cancer patients with distant metastasis. However, there is currently no predictive model for newly diagnosed Chinese colorectal cancer patients with distant metastasis, which means that the probability of outcome cannot be quantified.

Nomogram is a simple, multivariate visualization tool in which certain risk factors work together to predict and quantify the rate of the outcome of an individual patient (12). Therefore, in this study, we investigated clinicopathological factors in patients with colorectal cancer and aimed to develop a nomogram for predicting DM in newly diagnosed CRC patients. The results of this study will help to identify the high-risk groups of newly diagnosed colorectal cancer patients with DM according to the nomogram and help clinicians identify these patients early and choose appropriate treatment options, thereby improving prognosis and survival.

## Materials and methods

### Patients

The data included in the present study were obtained by two researchers at Zhong nan Hospital of Wuhan University and People’s Hospital of Gansu Province from January 2016 to December 2021. The inclusion criteria were as follows: 1) patients diagnosed with colorectal cancer for the first time from 2016 to 2021; 2) demographic variables, including age, sex, and body mass index (BMI), were available; 3) hematology test indicators, including hemoglobin, platelet count, and colorectal cancer tumor markers (including carcinoembryonic antigen (CEA), cancer antigen 125 (CA 125), and carbohydrate antigen 19-9 (CA 19-9)); 4) all newly diagnosed patients with colorectal cancer underwent colorectal tumor resection at first hospitalization, or patients with distant metastases underwent primary resection at least. Detailed pathological data (including tumor size, diameter, TNM stage, and histological grade) were obtained. 5) Newly diagnosed colorectal cancers diagnosed with distant metastasis should be confirmed by at least two imaging examinations or histopathological diagnoses. The exclusion criteria were as follows: 1) incomplete information, including demographic variables and hematology test indicators; 2) absence of important clinicopathological factors, such as grade, histological type, T stage, N stage, and M stage; 3) before obtaining pathological information, the patients underwent adjuvant therapy such as radiotherapy and chemotherapy; 4) patients with other malignant tumors (such as lung malignancies, hematological malignancies, and primary liver cancer). Finally, 327 patients were included to study the diagnostic risk factors of CRC patients with DM. Among them, 224 colorectal cancer patients from Zhong nan Hospital of Wuhan University were incorporated into the training set, and 103 colorectal cancer patients from Gansu Provincial People’s Hospital were incorporated into the testing set. In the present study, patients in the training set were used to develop the nomogram, and patients in the testing set were used to validate it. This study is a retrospective study and was conducted with the consent of the Ethics Committee of Zhong nan Hospital of Wuhan University. The ethics number is 2023019K.

### Statistical analysis

All statistical analyses in our present study were conducted with SPSS 26.0 and R software (version 4.2.0). Mean ± standard deviation (SD) was used to describe the quantitative data; number and percentage (N, %) were used to describe these categorical data. Student’s *t*- test was used to compare differences in continuous variables between groups if the variables followed a normal distribution. The χ^2^ test or Fisher’s exact test was used for categorical variables. In the present study, a *p*-value < 0.05 (two-sided) was considered statistically significant. Univariate logistic analysis was applied to identify DM-related factors. The variables with *p*- value < 0.05 in the univariate logistic analysis were included in the multivariate binary logistic regression analysis to determine independent risk factors of DM in initially diagnosed CRC patients. There are some indicators (including CEA level and platelet count), although the *p*- value >0.05 in multivariate analysis; they have important significance for the prognosis of colorectal cancer, which is also included to develop the nomogram. The predictive nomogram was developed by the “rms” package in R software, the “ROCR” package calculated the C-index, the “pROC” package calculated and plotted the receiver operating characteristic (ROC) curve, and the “rmda” package drew the calibration curve (CC)), decision curve analysis (DCA), and clinical impact curve. The ROC curve (13), C-index, and calibration curve were used to evaluate their performance. Moreover, DCA and clinical impact curve were also used to evaluate the stability of the model (14).

## Results and discussion

### Results

#### Clinical characteristics of the patients

According to inclusion and exclusion criteria, a total of 327 patients were included in this research: 224 colorectal cancer patients from Zhong nan Hospital of Wuhan University were incorporated into the training set, and 103 colorectal cancer patients from Gansu Provincial People’s Hospital were incorporated into the testing set. The clinical characteristics of 327 patients are shown in **Table 1**.

**Table 1:** Clinical and pathological features of patients diagnosed with CRC.

	Training set (224)	Validation set (103)
Age	56.99 ± 12.95	47.91 ± 12.05
Sex		
Female	96 (42.9%)	40 (38.8%)
Male	128 (57.1%)	63 (61.2%)
Year		
Before the COVID-19 pandemic	70 (31.25%)	85 (82.5%)
During the COVID-19 pandemic	154 (68.75%)	18 (17.5%)
BMI (kg/m^2^)	23.46 ± 3.16	22.23 ± 2.99
Hypertension		
Yes	64 (28.6%)	11 (10.7%)
No	160 (71.4%)	92 (89.3%)
Diabetes		
Yes	27 (12.1%)	4 (3.9%)
No	197 (87.9%)	99 (96.1%)
Smoking		
Yes	51 (22.8%)	15 (14.6%)
No	173 (77.2%)	88 (85.4%)
Drinking		
Yes	28 (12.5%)	15 (14.6%)
No	196 (87.5%)	88 (85.4%)
HGB (g/L)	115.90 ± 24.31	121.19 ± 27.59
PLT (10^9^/L)	246.56 ± 86.78	250.50 ± 110.84
Tumor markers^*^		
Positive	101 (45.1%)	56 (54.4%)
Negative	123 (54.9%)	47 (45.6%)
CEA (ng/ml)	51.32 ± 248.31	39.59 ± 140.83
Site		
Right	45 (20.1%)	20 (19.4%)
Left	50 (22.3%)	12 (11.7%)
Rectum	129 (57.6%)	71 (68.9%)
Size (cm)		
<5 cm	125 (55.8%)	59 (57.3%)
≥5 cm	99 (44.2%)	44 (42.7%)
Grade		
I	17 (7.6%)	4 (3.9%)
II	166 (74.1%)	73 (70.9%)
III	41 (18.3%)	26 (25.2%)
T stage		
T1–T2	40 (17.9%)	17 (16.5%)
T3–T4	184 (82.1%)	86 (83.5%)
N stage		
N0	102 (45.5%)	51 (49.5%)
N1–2	120 (53.57%)	52 (50.5%)
M stage		
M0	160 (71.4%)	70 (68%)
M1	64 (28.6%)	33 (32%)
Metastatic sites		
Liver	37 (16.52%)	19 (18.45%)
Lung	16 (7.14%)	7 (6.80%)
Peritoneum	10 (4.46%)	6 (5.82%)
Pelvic cavity	6 (2.68%)	6 (5.82%)
Other distant diseases	3 (1.34%)	2 (1.94)

CRC, colorectal cancer; BMI, body mass index; HGB, hemoglobin; PLT, platelet; CEA, carcinoembryonic antigen; CA 125, cancer antigen 125; CA 19-9, carbohydrate antigen 19-9.

^*^Tumor marker positive means hematologic CEA > 5 ng/ml, or CA 125 > 35 U/ml, or CA 19-9 > 37 U/ml.

#### Risk factors of distant metastasis in CRC patients and construction of predictive nomogram

The training set comprised 224 patients: 64 cases (28.6%) with DM at initial diagnosis and 160 cases (71.4%) without it (**Table 2**). The most common distant metastatic sites were the liver, lung, and peritoneum; some patients showed multiple- organ metastasis. For example, there were 37 patients with liver metastasis in the training set, accounting for 16.52% of the total population and 57.81% of the metastatic population. Through statistical analysis, the results showed that there were no significant differences in age (*p* = 0.662), sex (*p* = 0.096), body mass index (*p* = 0.590), hemoglobin level (*p* = 0.235), tumor size (*p* = 0.089), and T stage (*p* = 0.986) between non-metastatic colorectal cancer and metastatic colorectal cancer. Platelet (PLT) count, CEA, tumor markers, tumor site, lymph node stage, and histological grade (Grade) were statistically significant: PLT (*p* = 0.007), carcinoembryonic antigen (*p* = 0.028), tumor markers (*p* < 0.001), tumor site (*p* = 0.012), N stage (*p* < 0.001), and histological grade (*p* < 0.001). Notably, there was a statistically significant difference (*p* = 0.011) between the non-distant metastasis group and the distant metastasis group in the diagnosis year based on the COVID-19 epidemic. However, since the medical order and public life have gradually returned to normal, there is a bias in the variable of the year of diagnosis. To facilitate the subsequent use of the model, we did not include this variable in the formulation of the nomogram.

**Table 2:** Clinical and pathological features between distant and non-distant metastases of the training set.

	CRC without DM (N = 160)	CRC with DM (N = 64)	p
Age	57.23 ± 12.77	56.39 ± 13.47	0.662
Sex			0.096
Female	63 (39.4%)	33 (51.6%)	
Male	97 (60.6%)	31 (48.4%)	
Year			0.011
Before the COVID-19	58 (36.2%)	12 (18.8%)	
During the COVID-19	102 (63.7%)	52 (81.2%)	
BMI (kg/m^2^)	23.39 ± 3.24	23.64 ± 2.96	0.590
HGB (g/L)	117.12 ± 23.87	112.85 ± 25.33	0.235
PLT (10^9^/L)			0.007
<350	146 (91.2%)	50 (78.1%)	
≥350	14 (8.8%)	14 (21.9%)	
CEA	16.70 ± 101.50	137.86 ± 426.15	0.028
Tumor markers			<0.001
Negative	112 (70.0%)	11 (17.2%)	
Positive	48 (30%)	53 (82.8%)	
Tumor size (cm)			0.089
<5 cm	95 (59.4%)	30 (46.9%)	
≥5 cm	65 (40.6%)	34 (53.1%)	
Tumor site			0.004
Right	24 (15%)	21 (32.8%)	
Left	34 (21.3%)	16 (25%)	
Rectum	102 (63.7%)	27 (42.2%)	
T stage			0.986
T1–2	40 (25%)	0 (0%)	
T3–4	120 (75%)	64 (100%)	
N stage			<0.001
N0	97 (60.6%)	5 (7.8%)	
N1–2	63 (39.4%)	59 (92.2%)	
Grade			<0.001
Grade 1	16 (10%)	1 (1.6%)	
Grade 2	126 (78.8%)	40 (62.5%)	
Grade 3	18 (11.2%)	23 (35.9%)	

CRC, colorectal cancer; DM, distant metastasis; BMI, body mass index; HGB, hemoglobin; PLT, platelet.

**Figure 1:**
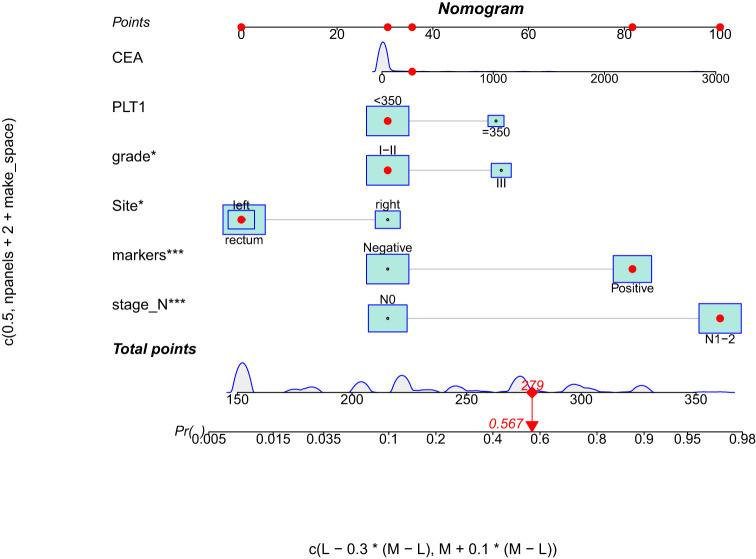
Nomogram for predicting DM from CRC patients. DM, distant metastasis; CRC, colorectal cancer.

To identify DM-related variables in CRC patients, 11 predictors were analyzed using univariate logistic analysis. The results revealed six predictors that were associated with DM in CRC patients, including PLT level (*p* = 0.009), CEA level (*p* = 0.032), histological grade (*p* < 0.001), colorectal cancer tumor markers (*p* < 0.001), N stage (*p* < 0.001), and tumor site (*p* = 0.005). Moreover, multivariate logistic analysis was performed on these six factors and showed that N stage (*p* < 0.001), histological grade (*p* = 0.038), and colorectal tumor markers (*p* < 0.001) were independent predictors for distant metastasis of colorectal cancer in newly diagnosed CRC patients (**Table 3**). Through the above three predictive factors (N stage, histological grade and colorectal tumor marker) and six predictive factors (PLT level, CEA level, tumor site, N stage, histological grade and colorectal tumor marker), the prediction models were established respectively, and it was found that there was no significant difference in C- index between the two models. Ultimately, based on the six DM-related variables, a diagnostic nomogram was developed for the risk assessment of DM in newly diagnosed CRC patients (**Figure 1**).

**Table 3:** Logistic analysis of risk factors of DM in training set CRC patients.

	Univariate (*p*)	OR	95% CI	Multivariate (*p*)	OR	95% CI
Age	0.660	0.99	0.97–1.11	–	–	–
Sex (male)	0.097	0.61	0.34–1.09	–	–	–
PLT (<350) (10^9^/L)	0.009	2.92	1.30–6.55	0.125	2.44	0.77–7.72
HGB (g/L)	0.235	0.99	0.98–1.01	–	–	–
Tumor markers (negative)	<0.001	11.24	5.41–23.38	<0.001	7.52	3.18–17.77
CEA (ng/ml)	0.032	1.00	1.00–1.01	0.406	1.01	0.99–1.00
N stage (N0)	<0.001	18.17	6.91–47.75	<0.001	15.48	5.07–47.32
Site (right)	0.005			0.059		
Left	0.145	0.54	0.23–1.24	0.048	0.30	0.09–0.99
Rectum	0.001	0.30	0.15–0.62	0.027	0.31	0.11–0.88
BMI (kg/m^2^)	0.589	1.03	0.94–1.13	–	–	–
Grade (I–II)	<0.001	4.43	2.18–8.98	0.038	2.55	1.05–6.21
Size (cm)	0.090	1.66	0.92–2.97	–	–	–

DM, distant metastasis; CRC, colorectal cancer; PLT, platelet; HGB, hemoglobin; CEA, carcinoembryonic antigen; BMI, body mass index.

#### Validation of training set for predictive nomogram

**Figure 2:**
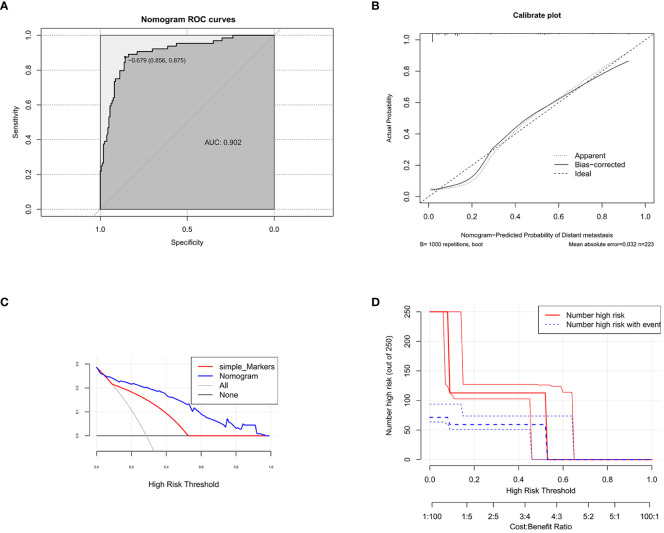
The receiver operating characteristic curve **(A)**, calibration curve **(B)**, decision curve analysis **(C)** (nomogram compared with tumor markers), and clinical impact curve **(D)** of the training set.

In the training set, we used ROC curves and C-index values to appraise the discrimination abilities of the nomogram. The C-index of the training set for predicting distant metastases was 0.902. The ROC curve of the training set was established, and the area under the curve (AUC) of the training set nomogram was 0.902 (95% CI, 0.857–0.948) (**Figure 2**). Furthermore, we also used a calibration curve, which is a novel method for appraising alternative prognostic instruments, and the DCA curve indicated that this nomogram can serve as an excellent diagnostic tool for DM in newly diagnosed CRC patients (**Figure 2**).

#### Validation of testing set for predictive nomogram

The testing set comprised 103 patients: 33 cases (32%) with DM at initial diagnosis and 70 cases (68%) without it. Similarly, univariate logistic analysis and multivariate logistic analysis were performed for six factors: platelet count, tumor markers, CEA, N stage, tumor site, and histological grade. The results showed that the platelet count (*p* = 0.010), tumor markers (*p* < 0.001), N stage (*p* < 0.001), tumor site (*p* < 0.001), and histological grade (*p* = 0.003) were statistically significant. Tumor markers (*p* = 0.001), N stage (*p* = 0.006), and tumor site (*p* < 0.001) were independent risk factors for distant metastasis of newly diagnosed colorectal cancer (**Table 4**). The statistical analysis results of the testing set were basically consistent with the results of the training set, which indicated that the six risk factors included in our study had good stability and universality, and the distant metastasis prediction model developed had high clinical practicability.

**Table 4:** Logistic analysis of risk factors of DM in the testing set CRC patients.

	Univariate (*p*)	OR	95% CI	Multivariate (*p*)	OR	95% CI
PLT (<350) (10^9^/L)	0.010	8.96	1.7–44.49	0.35	3.36	0.26–42.7
Tumor markers (negative)	<0.001	12.4	3.92–39.22	0.001	15.65	3.06–79.97
CEA (ng/ml)	0.225	1.002	1.00–1.01	0.351	0.999	0.994–1.00
N stage (N0)	<0.001	11.6	3.97–33.92	0.006	7.17	1.76–29.22
Site (right)	<0.001			<0.001		
Left	0.844	0.86	0.18–3.98	0.769	1.49	0.1–21.51
Rectum	<0.001	0.09	0.03–0.27	0.003	0.08	0.02–0.44
Grade (I–II)	0.003	4.16	1.63–10.6	0.119	3.36	0.73–15.43

DM, distant metastasis; CRC, colorectal cancer; PLT, platelet; CEA, carcinoembryonic antigen.

**Figure 3:**
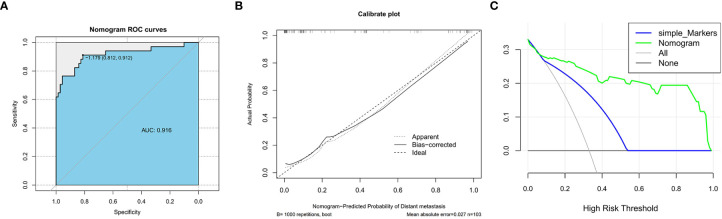
The receiver operating characteristic curve **(A)**, calibration curve **(B)**, and decision curve analysis **(C)** (nomogram compared with tumor markers) of the testing set.

According to the data of the testing set, we also established the ROC curve, calibration curve, and DCA curve. The AUC of the testing set nomogram was 0.916 (95% CI, 0.836–0.973). The calibration curve indicated good stability, and the DCA curve showed high net benefits of the diagnostic nomogram (**Figure 3**).

#### ROC curves for each risk factor in training set and testing set

**Figure 4:**
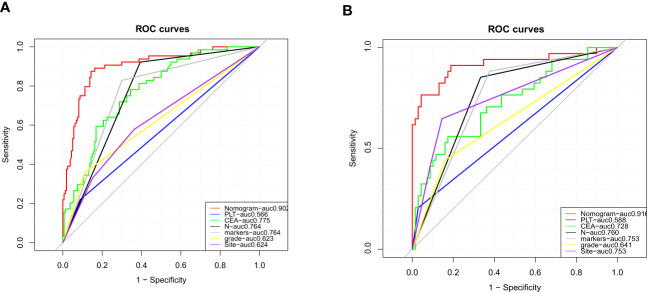
Comparison of area under the receiver operating characteristic curve between nomogram and each independent predictor in the training set **(A)** and the testing set **(B)**.

More importantly, the ROC curves of each predictor were also generated in both the training set and the testing set (**Figure 4**). In the testing set, the AUC was as follows: PLT count (AUC = 0.566), CEA (AUC = 0.775), histological grade (AUC = 0.623), colorectal cancer tumor markers (AUC = 0.764), N stage (AUC = 0.764), and tumor site (AUC = 0.624). In the testing set, the AUC was as follows: PLT count (AUC = 0.588), CEA (AUC = 0.728), histological grade (AUC = 0.641), colorectal cancer tumor markers (AUC = 0.753), N stage (AUC = 0.760), and tumor site (AUC = 0.753). The results showed that the AUC of all predictors alone was lower than the AUC of the nomogram, regardless of the training set or the testing set. In conclusion, the predictive diagnostic model can identify patients with a high risk of distant metastasis from newly diagnosed CRC patients.

### Discussion

This study retrospectively analyzed the clinical data of 327 patients with colorectal cancer (224 patients in the training set and 103 patients in the testing set), and the results showed that 98 patients (29.97%) (64 patients in the training set and 34 patients in the testing set) had developed distant metastases at the first visit, with an average age of 55 years. We found that platelet counts greater than 350 (10 * 10^9^/L), positive tumor markers, lymph node stage (N stage N1–N2), tumor histological grade (grade III), tumor location in the right colon, and high carcinoembryonic antigen concentration were associated with distant metastasis of colorectal cancer. Among these variables, tumor markers, lymph node stage, and histological grade were independent risk factors for distant metastasis.

In this study, we also found that patients with colorectal cancer who were first diagnosed during the COVID-19 epidemic had a higher risk of distant metastasis, which may be related to the delay in screening and diagnosis. The COVID-19 pandemic era impacted medical institutions/systems in various countries. The enormous diversion of medical resources toward SARS-CoV-2-dedicated wards dominated the clinical scenarios, with almost all planned public healthcare activities, including cancer screening, being suspended. A study in the United Kingdom (15) pointed out the detrimental effects on mortality of delaying diagnosis in symptomatic patients with CRC because of the SARS-CoV-2 pandemic. Recent data from Italy (16) also indicated that due to the impact of COVID-19, screening delays beyond 4–6 months would significantly increase advanced CRC cases and also mortality if lasting beyond 12 months. A large retrospective study from the *Journal of the American Medical Association* (*JAMA*) (17) also compared patients with colorectal cancer during the pandemic period and the prepandemic period, and the results showed that the SARS-CoV-2 pandemic was significantly associated with an increased rate of advanced-stage colorectal cancer.

Colorectal cancer is a common invasive tumor of the digestive system that is prone to distant metastasis. Metastases are a major driver of CRC-related mortality, with the liver and lung being the most frequently affected organs (18). Approximately 22% of colorectal cancer patients have distant metastases on their first visit to the hospital; meanwhile, the 5-year survival rate of these patients is less than 20%. For patients with resectable metastatic CRC, surgical resection of metastases is the only curative treatment option. For patients with unresectable metastatic CRC (3), the primary treatment is systemic therapy (including cytotoxic chemotherapy, biologic therapy such as antibodies to cellular growth factors, immunotherapy, and their combinations). Early treatment of patients with distant metastases can improve their survival rate. In some practical clinical features, the National Comprehensive Cancer Network (NCCN) guidelines said that TNM stage, age, tumor differentiation grade, vessel invasion, performance status, and tumor markers are important prognostic factors (19). Therefore, in this study, we established a nomogram based on clinical data and pathological features to predict the risk of distant metastasis in newly diagnosed CRC patients. The total score can be calculated by obtaining data on several easily accessible variables on the nomogram for each CRC patient. The risk of DM can then be easily identified on the nomogram, which will make the individualized clinical decision and clinical management more accurate.

The stratification theory of the left and right colon was proposed by American oncologist Bufill et al. in 1990 from the perspective of molecular genetics (20). Guideline (21) points out that the right side of the colon (cecum, ascending colon, and hepatic flexure) versus the left side of the colon (splenic flexure, descending colon, sigmoid, and rectosigmoid) and rectum represent a continuum of changes secondary to different embryological origins. Colorectal cancer is a heterogeneous malignant tumor with unique pathophysiological, anatomical, and clinical features. The location of tumor growth is an important factor affecting the progression, choice of treatment, and survival prognosis of colorectal cancer. Compared with that of the left colorectal tumor, the energy metabolism of the right colon tumor is mainly aerobic glycolysis of glucose, and tumor cells take advantage of aerobic glycolysis to decompose glucose and obtain energy (22). In terms of tumor histopathology, mucinous carcinoma, undifferentiated carcinoma, and sigmoidal ring cell carcinoma were the most common tumors on the right side of the colon, with high histological grade and low differentiation, while the left side of the colon was dominated by adenocarcinoma with medium and high differentiation (23). In molecular biology, BRAF, PI3KCA, and TGFBR2 gene mutations and heat shock protein regulation disorders are common in right colon tumors. Conversely, left colon tumors are often rich in KRAS gene mutations, HGFR/HER2 amplification, and high expression of amphiregulin and epithelial regulatory proteins (24). A systematic review in *JAMA* (25) also indicated that the side of the origin of CC (left *vs.* right) should be acknowledged as a criterion for establishing prognosis in both earlier and advanced stages of the disease. These show that right colon tumors are more invasive than left colon tumors.

Serological tumor markers are non-invasive and cost-effective indicators for the diagnosis, treatment, and prognosis of colorectal cancer. CEA and CA 199 are the two most common tumor markers used in colorectal cancer (26). The American Society of Clinical Oncology (ASCO) and the European Panel on Tumor Markers (EGTM) recommend CEA levels as a marker for follow-up after curative surgical resection of colorectal cancer. Rising levels indicate tumor recurrence after surgery or the development of metastatic disease (26). Some studies have also shown that an elevated preoperative CEA level is associated with a poorer prognosis and an increased risk of malignant tumor recurrence (27). Several other serological tumor markers, including CA 125, cancer antigen 72-4 (CA 72-4), and combined serum tumor biomarker levels, were positively correlated with tumor stage (28).

In this study, we found that three patients did not show definite metastases on preoperative imaging examination but were found to have metastases on imaging reexamination less than 10 days after surgery. Therefore, the risk factors selected by logistic regression analysis and the developed model can be used to quantitatively score whether each newly diagnosed colorectal cancer patient is at risk of distant metastasis and identify high-risk groups. 1) For high-risk patients without metastasis detected by the first imaging examination, clinicians need to further improve the evaluation of MRI (or PET-CT) and other imaging examinations, shorten the follow-up time of high-risk patients, and emphasize the importance of follow-up. 2) Clinicians should recommend molecular pathological testing for high-risk patients as early as possible. 3) For low-risk patients, the follow-up time can be appropriately extended to achieve individualized management for different patients.

However, several limitations to our study should be noted. First, this study is a retrospective study, which inevitably suffers from selection bias. Second, a limited number of patients (N = 327) included in this study may lead to possible errors. Therefore, follow-up studies need more prospective studies involving patients.

## Conclusions

Our study showed that N stage, grade, tumor markers, tumor site, preoperative CEA level, and platelet level were the risk factors for DM from CRC. N stage, grade, and tumor markers were the independent predictors. The nomogram we created may be a personalized, convenient, and more intuitive visualization tool for DM risk assessment in CRC.

## Preprint

A preprint has previously been published (https://doi.org/10.21203/rs.3.rs-2118512/v1) [17].

## Data availability statement

The original contributions presented in the study are included in the article/supplementary material. Further inquiries can be directed to the corresponding author.

## Ethics statement

This study is a retrospective study and was conducted with the consent of the Ethics Committee of Zhong nan Hospital of Wuhan University. The ethics number is 2023019K.

## Author contributions

J-HH collected the data, wrote the article, and analyzed the statistics. CC collected the data. YD, YY, Y-QL, and CW revised the article. YC provided fund support and revised the article. All authors contributed to the article and approved the submitted version.
